# Retraction Note: Association of severity of coronary artery disease by SYNTAX score (SS) and lower extremity arterial disease by duplex ultrasound (DUS) study—an Indian perspective

**DOI:** 10.1186/s43044-020-00130-9

**Published:** 2020-12-29

**Authors:** Saumen Nandi, Anindya Mukherjee, Dibbendhu Khanra, Kaushik Biswas

**Affiliations:** 1 Department of Cardiology, NRS Medical College, Kolkata, India; 2 Heart and Lung Centre, New Cross Hospital, Royal Wolverhampton NHS Trust, Heath Town, Wolverhampton, UK

**Retraction Note: Egypt Heart J 72, 56 (2020)**

**https://doi.org/10.1186/s43044-020-00091-z**

The Editor-in-Chief has retracted this article [1]. Following publication, the journal was notified that the published article reports an extension of study originally conducted between 1st May 2017 to 31st July 2018; the authors have confirmed that further ethics approval was not sought for the extension of the study.

All authors agree to this retraction.
